# Antibacterial and Cytocompatible pH-Responsive Peptide Hydrogel

**DOI:** 10.3390/molecules28114390

**Published:** 2023-05-27

**Authors:** Dona Imanga Upamadi Edirisinghe, Areetha D’Souza, Maryam Ramezani, Robert J. Carroll, Quenten Chicón, Cheyene L. Muenzel, Jonathan Soule, Mary Beth Browning Monroe, Alison E. Patteson, Olga V. Makhlynets

**Affiliations:** 1 Department of Chemistry, Syracuse University, 111 College Place, Syracuse, NY 13244, USA; 2 Biomedical and Chemical Engineering, Syracuse University, Bowne Hall, Syracuse, NY 13210, USA; 3 Department of Physics, Syracuse University, Syracuse, NY 13210, USA

**Keywords:** hydrogel, pH sensitive, antimicrobial, cytocompatible, self-healing, rheology, wound healing

## Abstract

A short peptide, FHHF-11, was designed to change stiffness as a function of pH due to changing degree of protonation of histidines. As pH changes in the physiologically relevant range, G′ was measured at 0 Pa (pH 6) and 50,000 Pa (pH 8). This peptide-based hydrogel is antimicrobial and cytocompatible with skin cells (fibroblasts). It was demonstrated that the incorporation of unnatural AzAla tryptophan analog residue improves the antimicrobial properties of the hydrogel. The material developed can have a practical application and be a paradigm shift in the approach to wound treatment, and it will improve healing outcomes for millions of patients each year.

## 1. Introduction

The pH of wounds changes during wound healing, going from pH ~5.5 when first made to pH ~8 when almost resolved [1]. This property of wounds motivates us to design a hydrogel material that adapts its stiffness to different pH values and could be used to adapt to different healing stages, thus facilitating the wound-healing process by releasing encapsulated fibroblasts at a reduced pH. The delivery of fibroblasts and keratinocytes has been used as a cell-based therapy for wound healing [2]. Other desirable characteristics of such materials would be self-healing ability and antimicrobial properties. Adaptable gels are shear-thin and flow under applied strain but recover their gel properties once the stress has been removed [3,4,5]. Self-healing hydrogels do not leak to neighboring tissues and can be delivered by injection, and they could re-anneal in the wound bed or in the destination site, providing a convenient form of application [6,7]. Antimicrobial hydrogels are especially popular as wound dressings because they provide both a moist environment and antimicrobial protection, resulting in improved healing outcomes [8,9,10,11]. Finally, the hydrogel material should be cytocompatible with the skin cells; therefore, we chose to use peptides for the hydrogel formation because they are generally cytocompatible [12,13,14,15,16]. The small size of the peptides gives them an advantage over natural materials [17] (such as alginate [18]) as they can be easily modified (e.g., via the introduction of non-canonical amino acids, RGD motif, etc.) [12,13,14,15,19,20,21].

Based on the above requirements, a hydrogel material suitable for wound healing applications was designed. The advantage of using peptide-based materials when compared to polymeric materials (natural or synthetic) is their ease of synthesis and incorporation of modifications (such as non-natural amino acids and RGD motif) and their cytocompatibility [12,13,14,15,19,20,21]. A synergistic combination of multiple properties is important because self-healing is essential for the delivery of the hydrogel via a syringe, and antimicrobial properties and cytocompatibility are critical for practical applications.

To create the hydrogel material, a peptide scaffold was used. Current peptide-based hydrogels lack some characteristics, as shown in Table S1. In contrast, our material combines all the characteristics necessary for an injectable wound dressing: self-healing, antimicrobial properties, cytocompatibility, and pH sensitivity. The peptide sequence was designed based on our previous results and the literature review. We had already successfully shown that a hydrogel material with Cu(II) can change the stiffness in response to copper reduction [22], and the peptide in that work served as the starting point of the design for this work. 

In this work, the first goal was achieved—the design of an antimicrobial, injectable, cytocompatible peptide hydrogel that is sensitive to changes in pH. The next step would be cell encapsulation and protection of fibroblasts during injection. Additionally, this work showed that having AzAla residues in the peptide sequence improves the antimicrobial properties of hydrogels. What makes our hydrogel system unique is a synergistic combination of stimuli responsiveness and antimicrobial peptides in a self-healing hydrogel, properties that are useful for wound healing applications [8,12,23,24,25,26,27,28,29]. Wounds from pressure ulcers [30], diabetic ulcers [31], surgery, or burns are serious global health issues that affect millions of patients worldwide [32], and our hydrogel material is suitable for the healing of wounds.

## 2. Results

**Figure 1:**
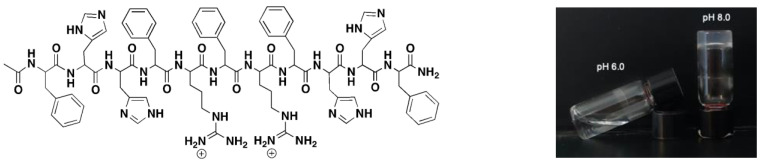
Sequence of pH sensitive peptide, namely, FHHF-11, and pH sensitivity of the peptide. The picture on the right was taken after the FHHF-11 peptide (1 wt%) was incubated with the buffer (100 mM HEPES, 100 mM MOPS, pH 6 or pH 8) for 15 min at 37 °C.

Peptide design: Based on our previous work and the data from the literature, the core of the peptide was designed to include phenylalanines and histidines. We also included two arginine residues to increase the antimicrobial properties of the peptide (Figure 1) [33,34,35,36]. The resulting peptide was shown to assemble into a hydrogel at pH 8 but not pH 6 (Figure 1). The presence of two arginine residues in the sequence should increase the bactericidal activity due to the cationic charge, which we demonstrate through the antimicrobial studies described in this work. Presumably, hydrogel dissipates at a lower pH due to the protonation of histidines and the increase in electrostatic repulsion. Therefore, the presence of histidines drives pH sensitivity as histidines have a pKa value of 6, below which it gets protonated, making the peptide backbone positively charged. Phenylalanine side chains impart hydrophobicity to the peptide and support the self-assembly through π–π stacking interactions leading to the formation of the hydrogel [37].

The sequence of the FHHF-11 peptide (Figure 1) was further modified to evaluate Arg to Lys substitution and the relative position of histidine. Due to a ~2 unit drop in the side chain pK_a_ of free amino acid, we envisioned that replacing Arg in the FHHF-11 sequence with Lys would result in peptide gelation at a lower and more physiological pH value. Changing just one Lys with Arg in MAX1 produced a more rigid gel at pH 8 [38]. We prepared the corresponding peptide but did not observe significantly different behaviors in gelation or pH sensitivity (Table 1, Figure S1). Removing terminal Phe residue or placing Phe between His resulted in no observed formation of hydrogel (Table S2). We conclude that peptides assemble into β-sheet fibers based on CD and TEM spectra and our previous results with Phe-containing sequences (Figure S2) [22]. 

Antimicrobial properties: In addition to having a peptide that assembles into a good hydrogel, we would like to create antimicrobial material. Generally, antimicrobial peptides are short, cationic, and amphiphilic molecules that evolved over time to selectively kill bacteria but be cytocompatible toward mammalian cells and show little potential for resistance development [26,39].

**Figure 2:**
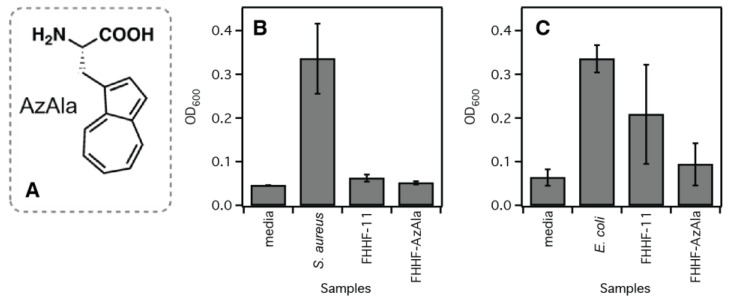
Incorporation of AzAla non-natural amino acid (**A**) improves the antimicrobial properties of the FHHF-11 peptide (**B**,**C**). FHHF-AzAla is FHHF-11 with AzAla amino acid. Experiments represent the average of eight wells and two independent preparations of AzAla peptides (synthesis and stock preparation).

We measured the antimicrobial properties of FHHF-11 hydrogel using both Gram-negative (*E. coli*) and Gram-positive (*S. aureus*) organisms and showed that hydrogel killed *S. aureus* organisms and, to a lesser extent, *E. coli* in contact with the material for 13 h (Figure 2). However, the effect of antimicrobial properties enhancement by incorporation of AzAla in the sequence was more pronounced in the case of *E. coli*. The fact that antibiotics are more efficient toward Gram-positive bacteria is well documented [40,41,42]. The contribution of arginines to antimicrobial properties was tested by using the FHHF-K sequence along with FHHF-11 (Figure S3), but unfortunately, we did not see an improvement of K over R in antimicrobial properties of peptide hydrogel. To further improve gelation and antimicrobial properties, we replaced Phe or Arg in the sequence with Trp. Peptides with tryptophan in the sequence have been reported to have better antimicrobial properties and better cytocompatibility [43,44,45]. We investigated the contributions of a cation-π interaction to the antibacterial activity of a newly designed peptide hydrogel. Cation-π interactions are a common feature of many antibacterial peptides, where they assist in the disruption of bacterial membranes. In Table S2, we compare the influence of tryptophan on the gelation properties of FHHF-11. Replacing Phe or Arg with Trp decreased the stiffness of the hydrogel and did not improve the antimicrobial properties of FHHF-11. However, FHHF-11 hydrogel (1 wt%) had improved antimicrobial properties when AzAla amino acid was incorporated (Figure 2), although the effect observed is not very significant. Non-natural amino acid Azulenyl-Alanine (AzAla) was developed as an analog of tryptophan [46]. Substitution of tryptophan by AzAla would allow studying the mechanism of action of AMPs by using unique properties of this amino acid, such as the ability to be excited separately from tryptophan in multi-Trp AMPs and environmental insensitivity. In our previous work, we found that the antimicrobial and bactericidal activity of the original peptide was preserved, while cytocompatibility with human cells and proteolytic stability was improved [47]. In this work, we investigate the effect of Trp → AzAla substitution on the formation of hydrogel and its antimicrobial properties (Table 1). 

**Figure 3:**
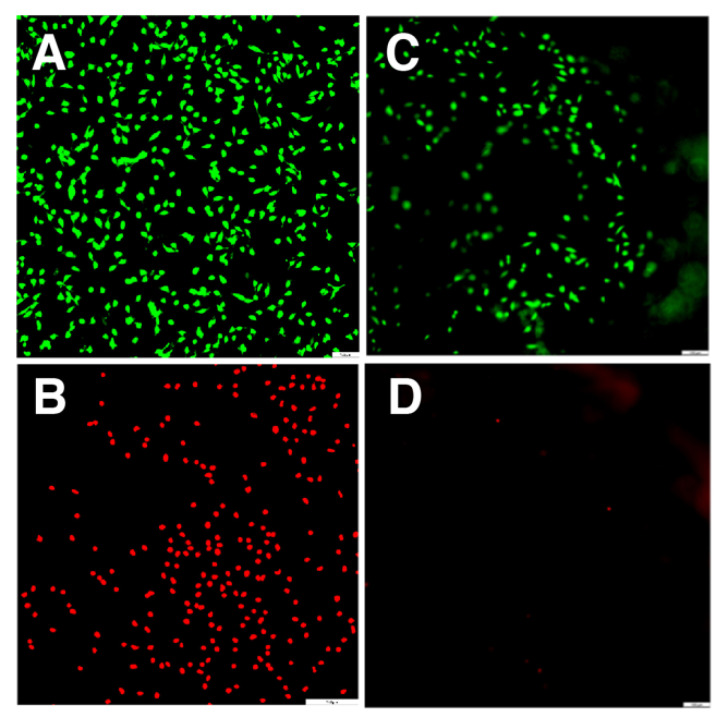
Live/dead assay for the mouse embryonic fibroblast cells: control with live (**A**) and dead cells (**B**) and fibroblasts on the surface of FHHF-11 hydrogel showing live (**C**) and dead (**D**) cells. The morphology of the gel is uneven and has a multiple number of planes. Incubation time of cells on hydrogel is 5 h at 37 °C. DMEM + FSB medium (pH 7.4) was used for the incubation.

Cytotocixity: The gels were tested for cytotoxicity against mouse embryonic fibroblasts (3T3, ATCC) since fibroblasts are a major group of cells that are considered to perform an important role in wound repair and healing [48]. The cytotoxicity was evaluated using live/dead assay, and the prepared gel was incubated with cells for 5 h to observe the cell attachment. Having a very low number of dead cells in the presence of the peptide (Figure 3) suggests that the hydrogel has very low toxicity towards mammalian cells. Next, we prepared the RGD derivative of the peptide and measured that it forms a good hydrogel material with higher cell attachment as compared to the parent sequence (Figure S4, Table 1). RGD [49,50,51], an integrin binding sequence present in ECM proteins, is widely utilized to enhance cell adhesion to mammalian cells [20,52,53,54,55,56,57]. The spacer length between RGD and hydrogel is very important for proper cell adhesion [58]. In this work, we showed that introduction of RGD linked with a single C-terminal glycine leads to some stiffness loss, but the peptide still assembles into the hydrogel. The placement of RGD and the linker is critical as it prevents hydrogel formation if it located at the N-terminus (our previous unpublished results). We used 1 wt% peptide to form a gel as this concentration of peptide was used throughout the manuscript; however, we are planning to use a higher concentration in the future to be able to evaluate peptide toxicity more precisely.

**Figure 4:**
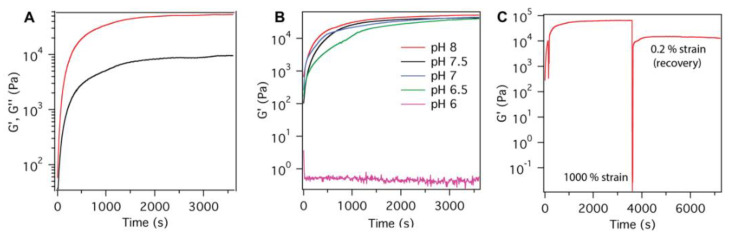
Rheology data for FHHF-11 hydrogel (1 wt%). (**A**) Evolution of storage modulus over time, where the red curve represents storage modulus (G′), and the black curve shows loss modulus (G″), pH 8.0. (**B**) The storage modulus (G′) evolution as a function of hydrogel pH. (**C**) The storage modulus during a self-healing test where the shear strain amplitude is increased from 0.2% strain to 1000% strain to examine the self-healing properties at 37 °C, 100 mM HEPES, pH 8, 285 μL).

**Figure 5:**
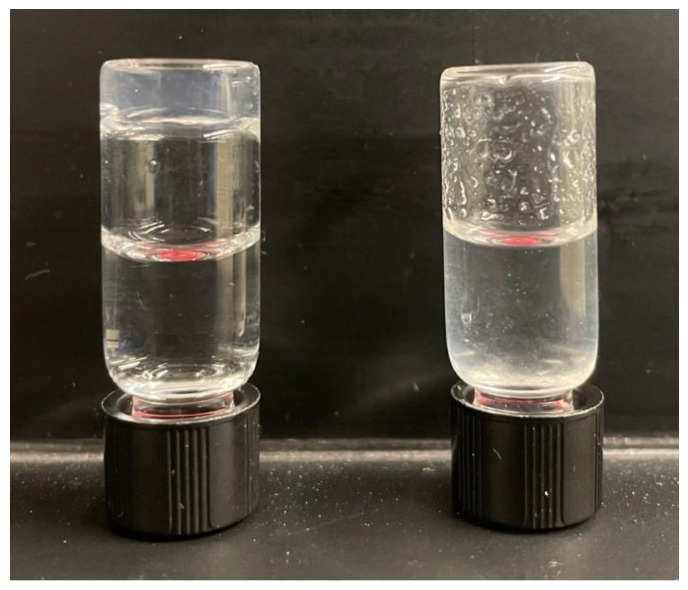
Dissolution of the hydrogel in buffer pH 6 takes only 10 min. The vial on the left is hydrogel in buffer pH 8 (remains stable hydrogel for hours), and on the right is shown the same hydrogel but in pH 6 buffer.

Rheology: Frequency sweep and strain sweep experiments were performed at 0.2% strain and 1 Hz frequency, respectively, to ensure that all subsequent rheology experiments were within the linear viscoelastic (LVE) regime (Figure S5). The storage modulus G′ (51.5 ± 0.5 kPa) was found to be greater than the loss modulus G″ (9 ± 1 kPa), which is a known property of a gel (Figure 4a). Hydrogels typically have a G′ that is about an order of magnitude greater than G″ [59]. A mixture of HEPES and MOPS buffers were used to cover a wide range of pH values and eliminate possible buffer effects (Figure 4b). The final G′ value of the hydrogel exhibited a decreasing trend upon decreasing the pH of the gel medium, with the gel failing to form at pH 6 (Table 1). This decrease can be correlated to the protonation of side chains of histidine residues at lower pH. Self-healing is essential for the delivery of the hydrogel via a syringe. Such adaptable gels are shear-thin and flow under applied strain but recover their gel properties once the stress has been removed [3,4,5]. The self-healing properties of the gel were tested by applying a large shear strain amplitude (1000% strain) for a short time before allowing the gel to recover for 1 h (Figure 4c). The gel exhibited 20% recovery from its initial G’ value of 65 kPa. An image of the hydrogel after 15 min self-healing is shown in Figure S6. To test the extent of self-healing, several cycles of repair were monitored at 0.2% strain after applying 1000% strain (1 h recovery) (Figure S7). The hydrogel appears to demonstrate self-healing in the tested range of pH (7–8), the pH range needed for self-healing in wound dressing. Lower pH values are expected to solubilize the gel and release encapsulated fibroblasts. We also evaluated the dissolution of hydrogel upon exposure to a buffer of low pH. In this experiment, we observed that for a hydrogel preformed at pH 8, the hydrogel quickly dissolved in pH 6 buffer but remained a stable hydrogel composite in pH 8 buffer (Figure 5). Finally, to test the hydrogels’ potential use in wound healing experiments, we evaluated the hydrogel stiffness in response to the presence of metal cations (Figure S8). For the cations Ca(II) and Mg(II) [60,61,62] at concentrations present in physiological fluids, we observed no change in the gelation of the material, even at high concentrations (6 mM final metal concentration). 

## 3. Discussion

We designed a peptide called FHHF-11 that assembles into a pH-sensitive, antimicrobial, cytocompatible, and self-healing hydrogel material. During the design, we showed that caps at the ends of the peptide sequence are important for gelation and placement of His in the sequence (Table 1). On C-term, we have an amide and N-term—acyl, and the presence of charges without caps present a material with 0 Pa and 940 Pa, respectively, thus decreasing gelation of the original peptide FHHF-11 significantly (down from ~50,000 Pa). We demonstrated that the hydrogel has properties needed for wound-healing applications. Here, we show that the RGD-modified hydrogel has improved cell attachment, demonstrating why peptide-based hydrogels have an advantage over polymeric materials. In addition to mentioned peptide advantages, the small size of the peptide and uniform composition of the hydrogel allow the characterization of the material by different experimental and theoretical methods, making peptide-based hydrogels ideal candidates for developing robust structure-activity-morphology relationships. With this advantage of peptides, we are going to study the mechanism of antimicrobial properties using already-developed methods. Peptides have before been used to manufacture pH-responsive hydrogel material, but antimicrobial and self-healing properties important for wound healing applications have not been shown [63]. Histidines have also been used to make self-assembling peptides sensitive to pH [64]. Therefore, using His as a tool to make material pH-responsive is based on solid precedent but also presents innovation in using much simpler peptide-based materials with antimicrobial properties. Here, we provide a review of other pH-sensitive peptide-based materials (Table S1). 

In this work, we created a pH-responsive material with suitable properties to create wound dressing. Next, we investigated the contributions of a cation-π interaction to the antibacterial activity of a newly designed peptide hydrogel. Peptides that include tryptophan in the sequence have better antimicrobial properties and better cytocompatibility [43,44,45]. Cation-π interactions are a common feature of many antibacterial peptides, where they assist in the disruption of bacterial membranes. In addition, previous work indicated that having Trp/Arg in the sequence helped with peptide gelation and antimicrobial properties [45]. We found that while incorporation of Trp did not improve the antimicrobial properties of our hydrogel material, Trp-analog, and AzAla did improve these antimicrobial properties. 

Our current work is focused on our self-healing hydrogel for cell protection during syringe delivery, as was shown previously for stem cells [65], cortical neurons [66], and fibroblast cells [67,68,69,70]. The material developed in the current work will combine pH-responsiveness and cell protection to deliver fibroblasts when the pH of the wound is low. Therefore, the dressing would adapt to healing stages and deliver cells when they are most needed. 

## 4. Materials and Methods

The Rink Amide resin was purchased from Chem-Impex International, and Fmoc-protected amino acids were purchased from Shanghai GL Biochem Ltd. The reagent *N*,*N*-Dimethylformamide (DMF, 99.9%) was purchased from Pharmco by Greenfield Global, and Piperazine was purchased from Chem-Impex International. Milli-Q water from Elix3 Millipore Sigma Water Purification System was used to prepare the samples and buffers. 

Peptide synthesis and purification: The peptide was synthesized following the manual Fmoc Solid-Phase Synthesis. Rink Amide resin (ChemImpex, Wood Dale, IL, USA) was swelled at 37 °C shaking at 100 rpm for 1 h, and the coupling of Fmoc-protected amino acids (GL Biochem Shanghai Ltd., Shanghai, China) was also performed at 65 °C. Arginines were double-coupled for 10 min at 65 °C, Lysine residues were coupled for 5 min at 65 °C, and AzAla was coupled for 40 min at room temperature. AzAla amino acid was prepared as previously described [47]. Peptide was acetylated using acetic anhydride mixed with *N*,*N*-Diisopropylethylamine, and DMF at the N-terminus at room temperature for 20 min. The peptide was cleaved from the resin, and the side chains were deprotected using the cleavage solution, which is a mixture of trifluoroacetic acid (TFA, 99.9%), H_2_O, and triisopropylsilane (99.78%) in a ratio of 95:2.5:2.5 by volume for 2 h at room temperature. The crude peptide was precipitated and washed with cold methyl-tert-butyl ether (99.9%). The identity of the peptide was confirmed by the MALDI-TOF mass spectrometry before and after purification (Bruker Microflex MALDI-TOF mass spectrometer (Billerica, MA, USA), Table S2). The peptide was purified using the preparative reverse-phase high-performance liquid chromatography system (Varian ProStar 210 (Palo Alto, CA, USA) preparative reverse-phase high-performance liquid chromatography system with a Jupiter 15 μm C4 300 A, LC column using a linear gradient of solvent A (0.1% TFA in Milli-Q water and solvent B (90% CH_3_CN, 9.9% Milli-Q water, and 0.1% TFA) with an injection volume of 5 mL and a flow rate of 20 mL/min, room temperature. The purity of the final peptide was evaluated using the analytical high-performance liquid chromatography system (Agilent Infinity II 1260 with an Analytical Zorbax (San Francisco, CA, USA) Eclipse XDB-C18 column (4.6 mm × 150 mm)). Peptide stock solutions were prepared from the lyophilized powder (above 90% purity). 

Peptide stock preparation: Pure lyophilized peptides were dissolved in ice-cold MilliQ water to make 12.1 mM stock solutions (2 wt% solution). The concentration of the peptide solution was determined using the UV-Vis spectrophotometer (Agilent (Santa Clara, CA, USA) 8453 UV−Vis spectrophotometer) by measuring the absorbance at 214 nm and ε_214_= 56,857 M**^−^**^1^ cm**^−^**^1^) [71]. The peptide stock solutions were aliquoted into 150 μL fractions and lyophilized. The lyophilized aliquots were then stored at −20 °C until further use. For AzAla peptide, the concentration of 12.1 mM was determined by measuring the absorbance at 342 nm and ε_342_= 4212 M**^−^**^1^ cm**^−^**^1^.

Rheology: The storage modulus G′ and loss modulus G″ were monitored for 1 h with a Malvern Kinexus rheometer using a 20 mm diameter parallel plate geometry and a gap height of 0.75 mm. A prepared aliquot of the powdered peptide was dissolved in 150 μL of ice-cold water and vortexed for 10 s to make a solution of 2 wt% peptide. The peptide solution was then centrifuged for 5 min at 6000 rpm to remove bubbles. To prepare gel for measurement of G′evolution, 150 μL of 100 mM Hepes, pH 8.0 was added to the peptide solution. For pH-dependent rheology, 150 μL of a mixture of 100 mM Hepes and 100 mM MOPS (pH 6.0/6.5/7.0/7.5/8.0) was added to the hydrogel solution and pipette mixed. The salt content was not monitored when changing the pH but it is known that salt only positively impacts the gelation properties of peptides containing positive charges in the sequence [72,73,74,75]. Finally, 285 μL of the total solution was pipetted onto the rheometer plate. Gelation was monitored by conducting a continuous oscillatory shear sweep at a constant 0.2% shear strain at a frequency of 6.28 rad/sec (1 Hz) for 1 h at 37 °C. Frequency sweep experiments were conducted at a constant shear strain of 0.2% over a range of frequencies from 0–300 rad/sec. Shear strain sweep experiments were performed at constant 1 Hz frequency over a range of shear strain amplitudes of 0.1–50% strain. The hydrogel samples were assembled by mixing 2 wt% peptide stock in water and buffer (100 mM Hepes, pH 8.0) and incubating at 37 °C for 1 h. The self-healing experiment was done by changing the shear strain from 0.2% to 1000% under a constant frequency of 1 Hz for a period of 30s time, followed by recovery of G′ at 0.2% strain for 1 h. 

Cytotoxicity experiment: Cytotoxicity experiments were conducted using live/dead assay. The lyophilized peptide (2 wt%, 75 μL stock) was dissolved in 75 μL of sterile Milli-Q water and placed in 3 wells (25 μL each) of a 96-well plate. The hydrogel was prepared by adding buffer (25 μL of 100 mM HEPES, pH 8.0) to each well containing peptide in water. Hydrogel samples were incubated at 37 °C overnight. The cultured mouse embryonic fibroblast cells (3T3, ATCC) with the medium DMEM (Dulbecco’s Modified Eagle Medium) supplemented with 10% fetal bovine serum (FBS) and 1% penicillin-streptomycin (P/S, Gibco) were seeded into the hydrogel samples at a concentration of 1 × 10^4^ cells/well and incubated at 37 °C/5% CO_2_ for 5 h (pH of the medium was 7.4). The cells with the culture medium were used as the positive control, while the cells treated with 50 μL of 30% H_2_O_2_ were used as the negative control. The medium was removed and washed with 1× PBS (200 μL). Then, the samples were treated with 50 μL of 2 μM solution of Live/Dead^®^ assay (live: Calcein AM, green; dead: BOBO-3 Iodide, red, Thermo Fisher Scientific) in each well and incubated for 20 min. During this process, samples were wrapped in aluminum foil prior to the beginning of the fluorescence imaging. The cells were imaged (3 field views/images) on a fluorescence microscope using blue and green light on a Leica DMI 6000B microscope (Wetzlar, Germany).

Antimicrobial studies: The hydrogel samples (70 µL of 1 wt% stock in 50 mM HEPES, pH 8) were prepared by mixing an equal volume of peptide stock in water (2 wt%) and buffer (100 mM Hepes, pH 8). The samples were placed inside 96-well plate (Greiner Bio-one), covered by BreathEasy membrane (Sigma-Aldrich), and incubated at 37 °C without shaking for 6 h. The culture of *E. coli* (ATCC 25922) and *S. aureus* (ATCC 25923) were started by diluting glycerol stocks of cells in LB (Luria Bertani, prepared ourselves ) and TSB (Tryptic Soy Broth, Becton, Dickinson, and Company) using 20 µL of *E. coli* stock in 5 mL of LB (first dilution). Next, the culture was further diluted using 20 µL of *E. coli* (first dilution) and 5 mL of fresh LB (second dilution), and 30 µL of *S. aureus* in 5 mL TSB (first dilution). Finally, the culture was further diluted using 30 µL of S. aureus first dilution and 5 mL of fresh TSB (second dilution). After incubation at 37 °C with shaking at 150 rpm for 5 hr, the second dilution of *E. coli* culture grew to OD_600_ = 0.8, and the first dilution of *S. aureus* culture grew to OD_600_ = 1.1. Both cultures were diluted to OD_600_ = 0.1 and allowed to grow for 1 h, after which the *E. coli* culture grew to OD_600_ = 0.32, and *S. aureus* grew to OD_600_ = 0.29. Both cultures were diluted to OD_600_ = 0.001 separately, and 200 µL of this culture was introduced above each sample, then the plate was covered with a new Breath Easy membrane. After 13 h incubation at 37 °C and 150 rpm, aliquots (100 µL) of each culture in contact with samples were analyzed for OD_600_ using a plate reader (Thermo Labsystems, Multiscan Spectrum). Control samples with culture were prepared by mixing water and buffer instead of peptide solution and buffer, followed by the culture with OD_600_ = 0.001 being introduced into the wells. ‘Media’ samples were prepared by mixing water and buffer followed by sterile LB medium or TSB medium addition to each well. Data represent averages of eight wells and two independent preparations of AzAla-containing FHHF-11.

Circular dichroism spectroscopy: The CD spectra were acquired on the Jasco J-715 CD spectrometer by collecting ten scans (4 s averaging time) for each spectrum and using a quartz cuvette with 0.1 mm path length. The measurements were performed on samples containing 0.25 wt% FHHF-11 peptide (3 mM) in buffer pH 6.0 and 8.0 (12.5 mM HEPES, 12.5 mM MOPS). The hydrogel samples were aged at 37 °C overnight to ensure gel formation before measurements. Care was taken that the sample absorbance never exceeded 1.5 at all wavelengths to produce reliable ellipticity values. Mean residue ellipticity (MRE, deg * cm^2^ * dmol**^−^**^1^) values were calculated using the following equation, where θ is ellipticity (mdeg), l is pathlength (cm), C is peptide concentration (M), N is number of residues.

$$\mathrm{MRE}=\frac{\theta }{10*C*l*N}$$

Transmission electron microscopy: FHHF-11 hydrogel samples (6 mM, 1%wt/v) in 50 mM HEPES 50 mM MOPS of pH 6 and 8 were prepared and incubated at 37 °C overnight to be aged. After preparation, 3 μL aliquots were applied to carbon-coated 200-mesh copper grids (Ted Pella) and left for 2 min. Excess liquid was wicked away with filter paper, and grids were then stained with one 5 μL drop of 2% (*w*/*v*) uranyl acetate, followed by immediate blotting. A second 5 μL drop of 2% (*w*/*v*) uranyl acetate was applied and left on the grid for 20 s. This was then blotted away, and grids were stored under a vacuum until imaging. Samples were viewed with a JEOL-JEM 2100F Field Emission electron microscope (Tokyo, Japan) at an acceleration voltage of 200 kV. Electron micrographs were recorded on a Gatan OneView 4K CCD camera (Pleasanton, CA, USA).

### Solubilization of Hydrogel

Hydrogel samples (300 μL) containing 1 wt% (6 mM) FHHF-11 in buffer (final 50 mM HEPES, pH 8) were formed in two glass vials and kept overnight at 37 °C. To check the pH-dependent solubility of the hydrogels, 900 µL of buffer (50 mM HEPES, pH 8) was added to one of the vials, and 900 µL of buffer (50 mM MOPS, pH 6) was added to the second vial. Both vials were swirled for 10 min. The vials were inverted to check the solubility of the hydrogels. The hydrogel in the buffer at pH 8 remained stable for about 15 h.

## 5. Conclusions

In this work, we have successfully created a peptide-based hydrogel that changes its stiffness as a function of pH, being liquid below pH 6 and forming the stiffest hydrogel in the physiological range (pH 7–8). By introducing both antimicrobial and self-healing properties into the hydrogel scaffold, we were able to create cytocompatible, antimicrobial, stimuli-responsive hydrogel materials for wound healing. This work opens the path to new approaches in pH-dependent fibroblast delivery. 

## Figures and Tables

**Table 1:** Rheological properties of hydrogels (1 wt% of peptides) measured at 0.2% strain, 37 °C after 1 hr. All runs were done in triplicates using 50 mM HEPES and 50 mM MOPS in the pH range 6–8, and the error bars represent standard deviation. Experiments at pH 8 were done in the following buffer concertation: 50 mM Hepes, pH 8 (final concentration).

Peptide Name	pH	Peptide Sequence	Shear Modulus G′ (Pa)
FHHF-11	8	Ac-FHHFRFRFHHF-CONH_2_	51,466 ± 4800
	7.5		42,000 ± 6850
	7		40,100 ± 2250
	6.5		35,350 ± 4950
	6		0.7 ± 0.2
FHHF-K	8	Ac-FHHFKFKFHHF-CONH_2_	94,000 ± 9000
	7.5		89,200 ± 28,800
	7		67,050 ± 10,550
	6.5		25,350 ± 10,750
	6		0.3 ± 0.1
No-Ac FHHF-11	8	NH_2_-FHHFRFRFHHF-CONH_2_	940 ± 340
COOH FHHF-11	8	Ac-FHHFRFRFHHF-COOH	0
HHF	8	Ac-HHFRFRFRFHH-CONH_2_	30
HFH	8	Ac-HFHFRFRFHFH-CONH_2_	65
FHFH	8	Ac-FHFHFRFRFHFHF-CONH_2_	60
FHHF-GRGD	8	Ac-FHHFRFRFHHFGRGD-CONH_2_	2620 ±360
W substitutions	8	Ac-WHHFRFRFHHF-CONH_2_	865 ± 126
	8	Ac-FHHFRFRFHHW-CONH_2_	125 ± 75
	8	Ac-FHWFRFRFHHF-CONH_2_	19 ± 9
	8	Ac-FHHFRWRFHHF-CONH_2_	5361 ± 3448
	8	Ac-FHHFKWKFHHF-CONH_2_	4140 ± 2356
	8	Ac-FHHFRFWFHHF-CONH_2_	435 ± 339
	8	Ac-FHHFWFRFHHF-CONH_2_	205 ± 168
AzAla substitutions	8	Ac-FHHFR(AzAla)RFHHF-CONH_2_	10,520 ± 3790

## Data Availability

Data is contained within the article or Supplementary Material.

## Supplementary Materials

The following supporting information can be downloaded at: https://www.mdpi.com/article/10.3390/molecules28114390/s1. References [63,64,76,77,78,79,80,81,82,83,84,85,86,87,88] are cited in the supplementary materials.

## Sample Availability

Samples of the peptides are available from the authors.
