# Supplementary material for: Antibacterial and Cytocompatible pH-Responsive Peptide Hydrogel
Source: Molecules. 2023 May 27;28(11):4390. doi: 10.3390/molecules28114390 (PMC10254169; doi:10.3390/molecules28114390)
Supplement: Supplementary file 1 [file molecules-28-04390-s001.zip › molecules-2289369-supplementary.pdf]

Table S1. pH-sensitive peptide-based hydrogels designed previously.

peptide	G′ (pH), wt%	Self-healing	Antimicrobial properties	Potential application	cytocompatibility	Reference
CKIKIKIK–I^{D}PPT–KIOIKIKC– NH_2	1500 Pa (7.4), 1.5 wt%	Yes	–	Anti-tumor drug delivery	Yes	1
EKAIQIAF	1500 Pa (7.4), 2 wt %	–	–	Anti-tumor drug delivery	Yes	2
SSSGGK*GGHHH Where * = palmitoyl tail	3500 Pa (9.0), 0.2 wt%	–	–	3D tissue scaffold	Yes	3
Lauryl-FFAGHH	–	–	–	–	–	4
FEFEFRFK	700 Pa (7.4), 2 wt%	–	–	Anti-tumor drug delivery	Yes	5
Nap-FEFK	10000 Pa (7), 2.6 wt%	Yes	–	Cell-supporting scaffold	Yes	6
VKVKVOVK–V^{D}PPT–KVEVKVKV	4000 Pa (7.4), 1.5 wt%	Yes	–	Anti-tumor drug delivery	Yes	7
Palmitoyl- VVAEEE	–	–	–	Anti-tumor activity	Yes	8
VKVKVKVK-V^{D}PPT-KVKVKVKV	1200 Pa (9), 2 wt%	–	–	–	–	9
LKELAKV LHELAKL VKEALHA	350 Pa, 0.35 wt%	–	–	Tissue engineering	Yes	10
(KIGAKI)3-TDPPG-(KIGAKI)3	13-20 Pa at 20°C, 0.5 wt%	–	Yes	Tissue engineering	–	11
FHHFRFRFHHF	~50,000 Pa (8), 1 wt%	Yes	Yes	Wound healing, drug delivery	Yes	This work

– Not shown

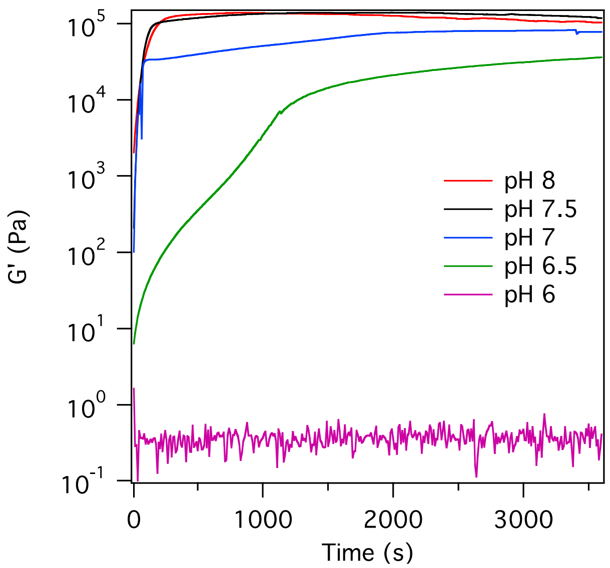

Figure S1. Rheology runs on FHHF-K peptide showing traces at different pH values. The hydrogel samples were assembled to make a final 1 wt% peptide in buffer 50 mM Hepes and 50 mM MOPS (pH 6.0/6.5/7.0/7.5/8.0) and running at 37°C for 1 hr.

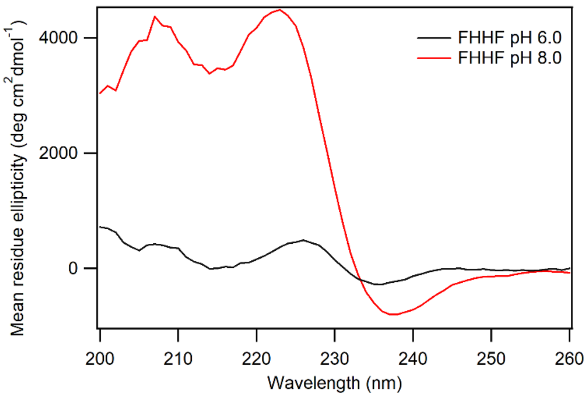

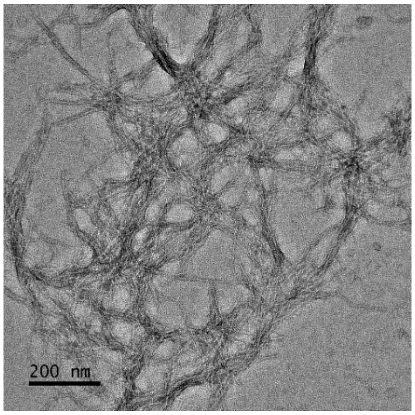

Figure S2. Peptide FHHF-11 assembles into fibrils at pH 8 as can be seen by CD (left) and TEM (right). Left figure shows CD spectra of FHHF-11 peptide in buffer mixture at pH 6 and 8. The positive signal at 210-230 nm is due to exciton coupling of Phe residues.[12-15] Samples were prepared by incubating FHHF-11 peptide (3 mM, 0.25 wt%) in buffer (12.5 mM HEPES 12.5 mM MOPS, pH 6 and 8) overnight at 37°C. CD spectra were collected with the pathlength of 0.1 mm and 10 scans for each sample.

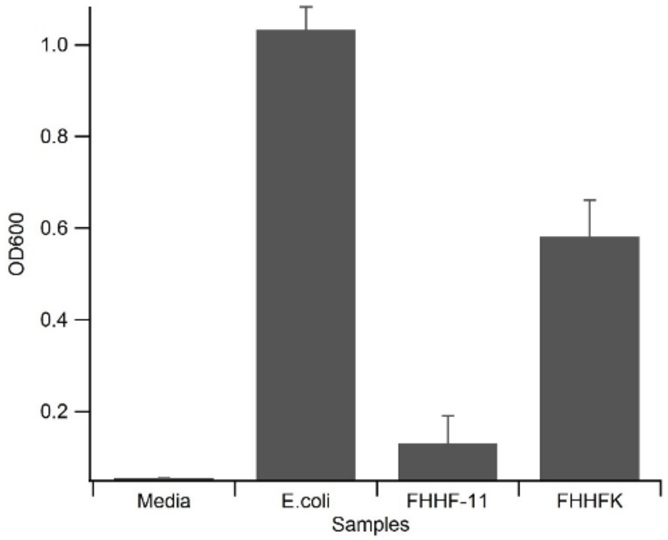

Figure S3. Inhibition of bacterial growth. Hydrogel samples were formed from 1 wt%. All samples except the first one (media) contained *E. coli* culture.

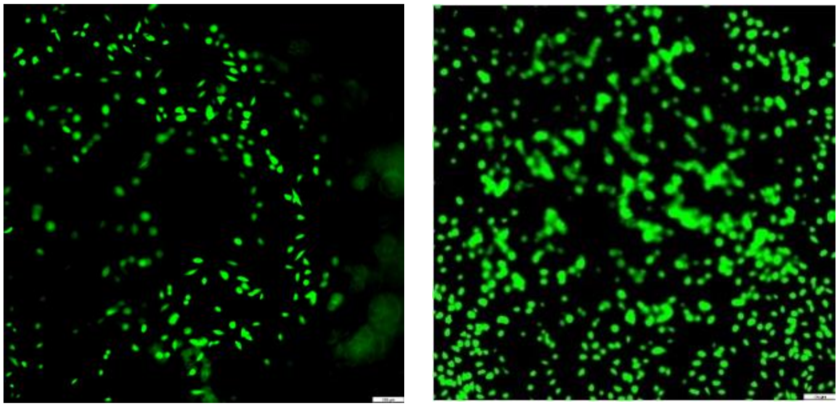

Figure S4. Live/dead assay for the fibroblast cells: fibroblasts on the surface of FHHF-11 hydrogel (left) and its GRGD derivative (right) showing live cells.

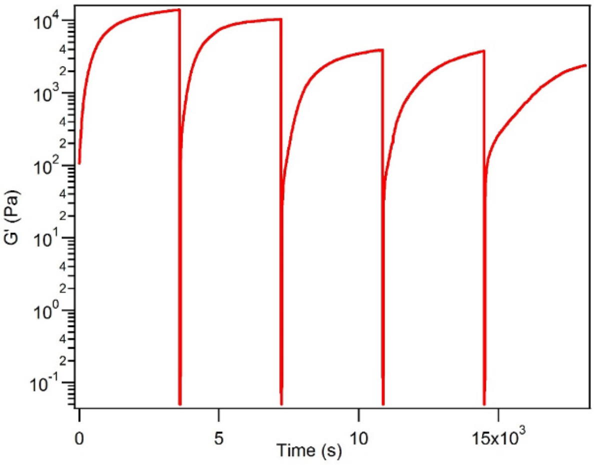

Figure S5. Shear recovery of the FHHF11 hydrogel prepared by mixing 1 wt% peptide FHHF-11 in buffer (50 mM HEPES/50 mM MOPS, pH 7). In each cycle the hydrogel was subjected to 1000% strain at 6.283 rad/s for 30 s at 37°C, followed by an oscillation time sweep experiment (0.2% strain) for 1 hr to check the sample's recovery after shear.

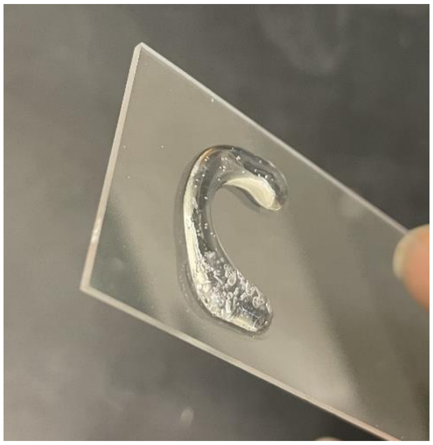

Figure S6. Recovery of hydrogel material after syringe delivery. 1wt% of peptide hydrogel was assembled inside a syringe (900 µl). Hydrogel was assembled by mixing 450 uL of peptide solution in water (2 wt%) with 450 uL of buffer (100 mM Hepes, pH 8) inside a syringe. The mixture was incubated for 15 min at 37°C and then the drawing was made on a glass slide, which was subsequently incubated at 37°C for another 15 min (a beaker with water was placed inside incubator).

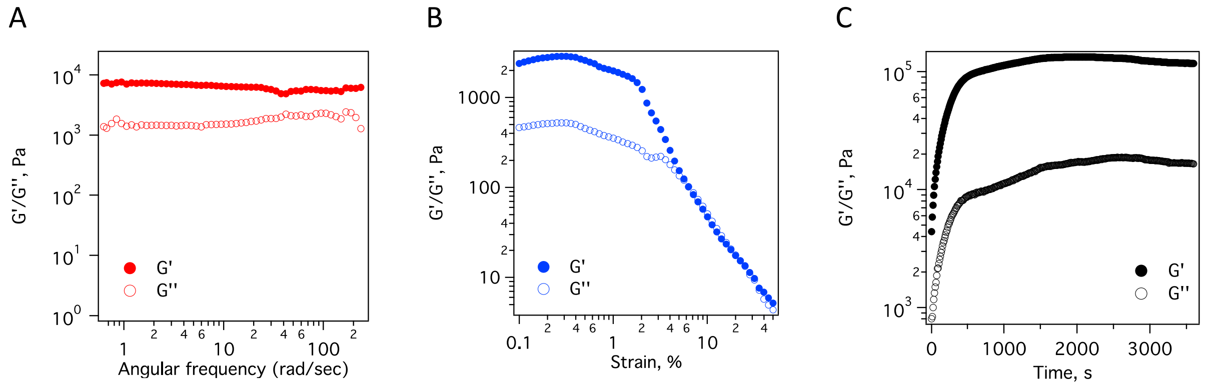

Figure S7. (A) Frequency and (B) strain sweep rheological data collected at 0.2% strain and 6.28 rad/s frequency (1 Hz) on the pre-formed gels. Closed and open symbols represent storage (G′) and loss (G′′) modulus. (C) The hydrogel samples were assembled by mixing 2 wt% peptide stock in water and buffer (100 mM Hepes, pH 8) and incubating at 37˚C for 1 hr.

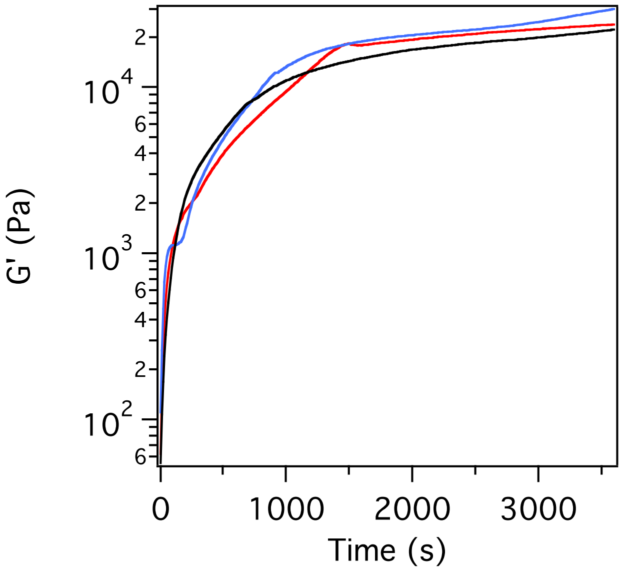

Figure S8. Rheology runs with FHHF-11 peptide at pH 8 without (black) and with Ca(II) (red) and Mg(II) (blue). The hydrogel samples without metal were assembled by mixing 2 wt% peptide stock in buffer (50 mM Hepes, pH 8) and running at 37°C for 1 hr. The hydrogel samples with metal ions were assembled by mixing 2 wt% peptide with 1 eq of metal in water and then the buffer (100 mM Hepes, pH 8) was added and samples analyzed by running at 37˚C for 1 hr.

Table S2. MALDI spectra of peptides used in this work.

Peptide name	Peptide sequence	Expected m/z	Observed m/z	MALDI spectrum
FHHF-11	Ac-FHHFRFRFHHF-$CONH_2$	1656	1656	Figure S9
No-Ac FHHF-11	NH_2-FHHFRFRFHHF-$CONH_2$	1613	1613	Figure S10
COOH FHHF-11	Ac-FHHFRFRFHHF-COOH	1657	1657	Figure S11
FHHF-K	Ac-FHHFKFKFHHF-$CONH_2$	1600	1600	Figure S12
HHF	Ac-HHFRFRFRFHH-$CONH_2$	1663	1660	Figure S13
HFH	Ac-HFHFRFRFHFH-$CONH_2$	1656	1656	Figure S14
FHFH	Ac-FHFHFRFRFHFHF-$CONH_2$	1951	1952	Figure S15
FHHF-GRGD	Ac-FHHFRFRFHHFGRGD-$CONH_2$	2040	2040	Figure S16
W substitutions	Ac-**W**HHFRFRFHHF-$CONH_2$	1695	1695	Figure S17
	Ac-FHHFRFRFHH**W**-$CONH_2$	1695	1695	Figure S18
	Ac-FH**W**FRFRFHHF-$CONH_2$	1705	1705	Figure S19
	Ac-FHHFR**W**RFHHF-$CONH_2$	1695	1695	Figure S20
	Ac-FHHFK**W**KFHHF-$CONH_2$	1638	1638	Figure S21
	Ac-FHHFRF**W**FHHF-$CONH_2$	1686	1686	Figure S22
	Ac-FHHF**W**FRFHHF-$CONH_2$	1686	1686	Figure S23
AzAla substitutions	Ac-FHHFR(**AzAla**)RFHHF-$CONH_2$	1705	1705	Figure S24

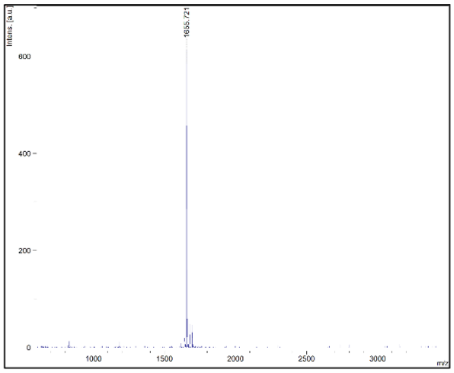

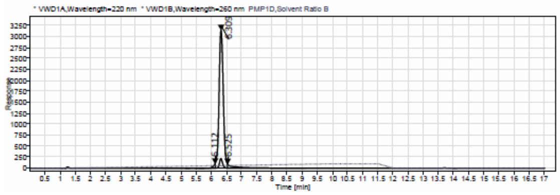

Signal: * VWD1A,Wavelength=220 nm

RT [min]	Width [min]	Area	Height	Area%
6.112	0.06	345.98	100.03	1.06
6.309	0.15	30567.81	3155.17	94.01
6.525	0.29	1602.06	92.24	4.93
	Sum	32515.86		

Figure S9. MALDI (left) and HPLC chromatogram (right) spectra of FHHF-11.

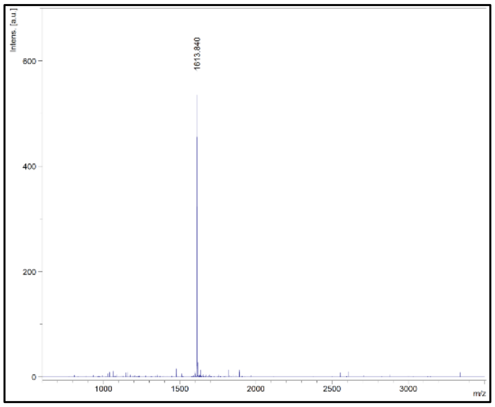

Figure S10. MALDI spectrum of No-Ac FHHF-11 (NH_2-FHHFRFRFHHF-$CONH_2$) peptide.

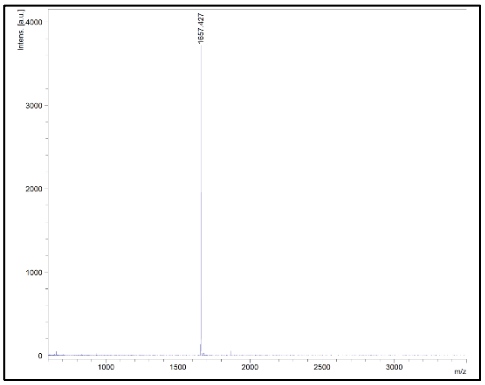

Figure S11. MALDI spectrum of COOH FHHF-11 (Ac-FHHFRFRFHHF-COOH) peptide.

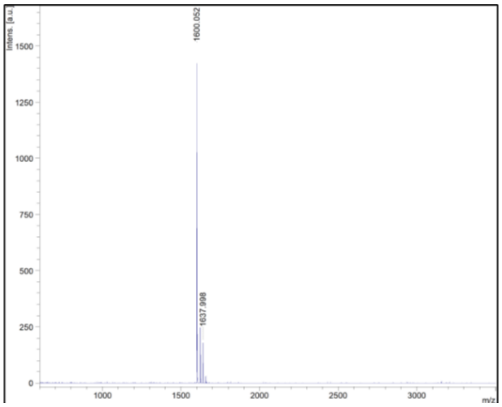

Figure S12. MALDI spectrum of FHHF-K (Ac-FHHFKFKFHHF-$CONH_2$) peptide.

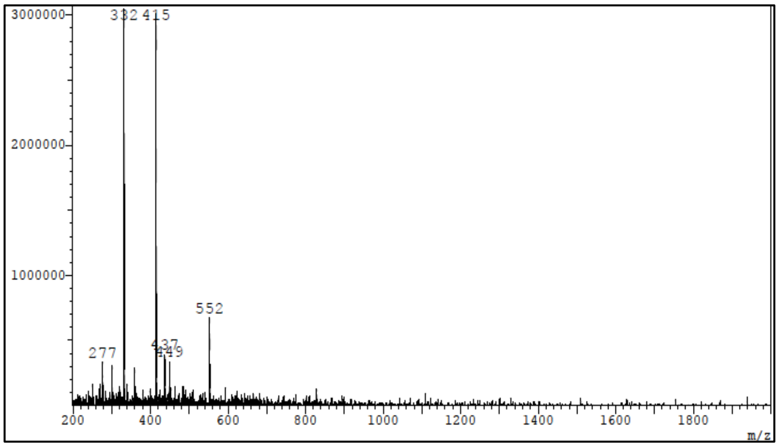

Figure S13. LCMS of HHF (Ac-HHFRFRFRFHH-$CONH_2$) peptide.

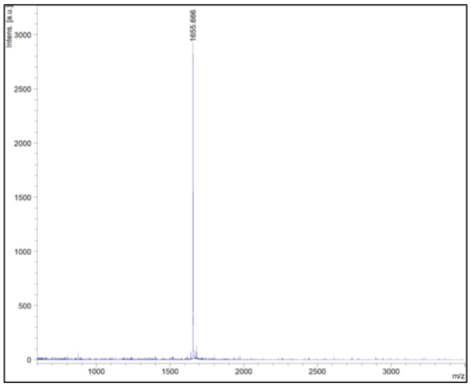

Figure S14. MALDI spectrum of HFH (Ac-HFHFRFRFHFH-$CONH_2$) peptide.

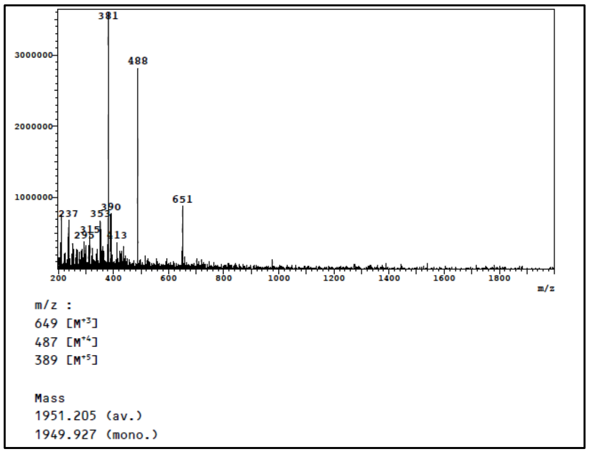

Figure S15. LCMS of FHFH (Ac-FHFHFRFRFHFHF-$CONH_2$) peptide.

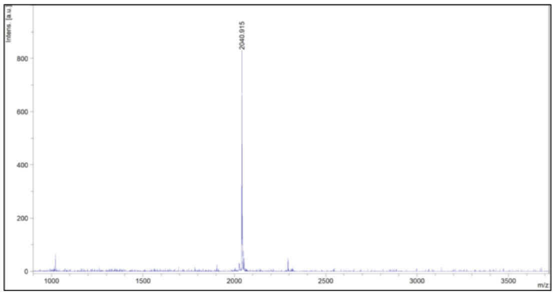

Figure S16. MALDI spectrum of FHHF-GRGD (Ac-FHHFRFRFHHFGRGD-$CONH_2$) peptide.

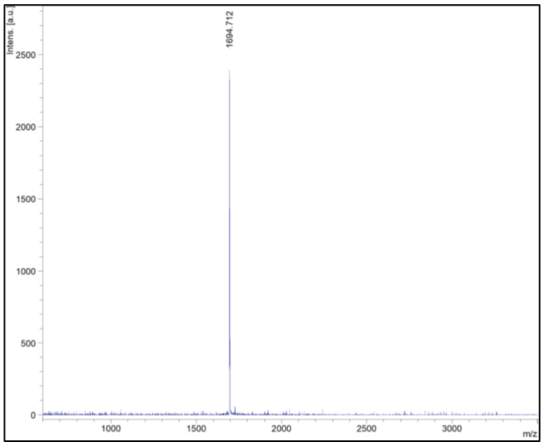

Figure S17. MALDI spectrum of Ac-WHHFRFRFHHF-$CONH_2$ peptide.

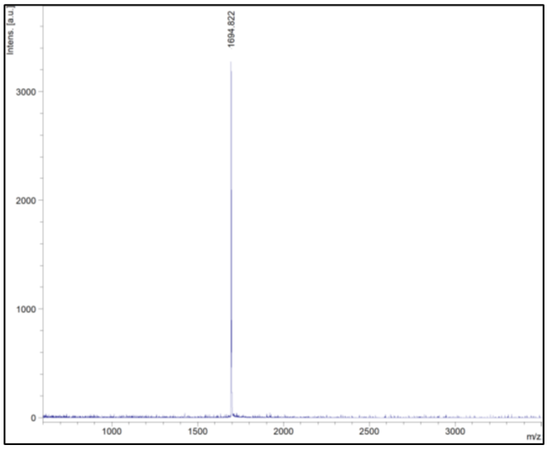

Figure S18. MALDI spectrum of Ac-FHHFRFRFHHW-$CONH_2$ peptide.

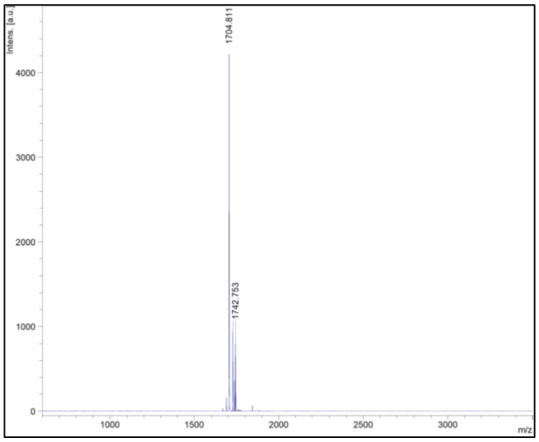

Figure S19. MALDI spectrum of Ac-FHWFRFRFHHF-CONH2 peptide.

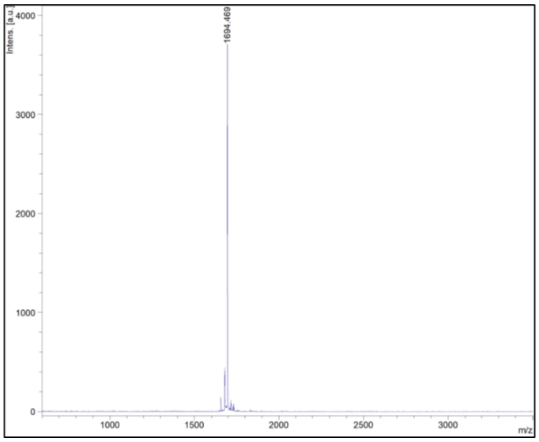

Figure S20. MALDI spectrum of Ac-FHHFRWRFHHF-CONH2 peptide.

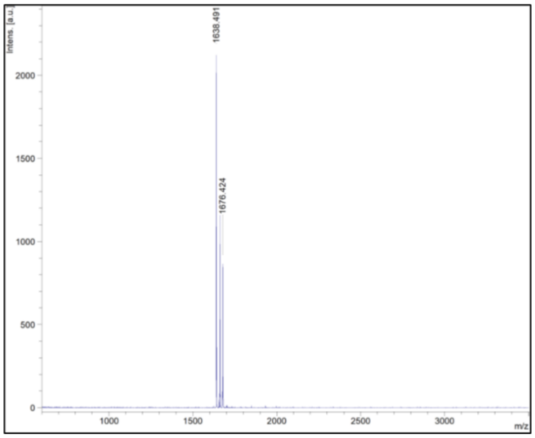

Figure S21. MALDI spectrum of Ac-FHHFKWKFHHF-CONH2 peptide.

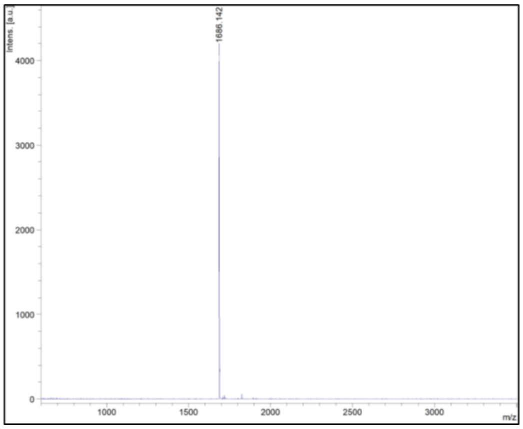

Figure S22. MALDI spectrum of Ac-FHHFRFWFHHF-CONH2 peptide.

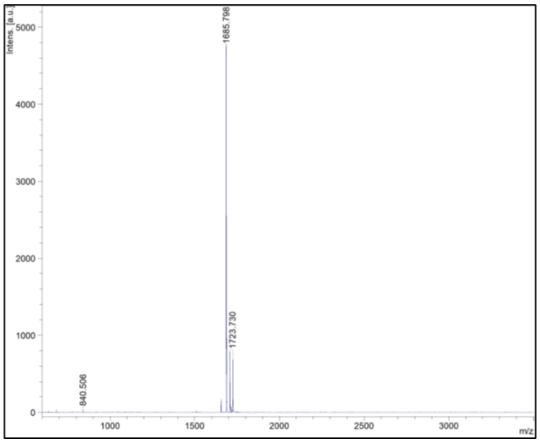

Figure S23. MALDI spectrum of Ac-FHHFWFRFHHF-CONH2 peptide.

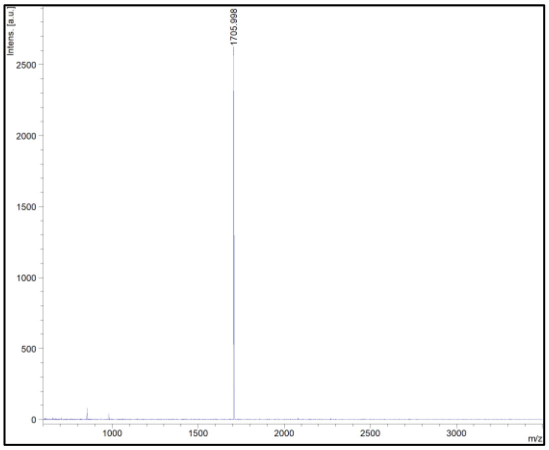

Figure S24. MALDI spectrum of Ac-FHHFR(AzAla)RFHHF-CONH2 peptide.

References:

1. Zhu, Y.; Wang, L.; Li, Y.; Huang, Z.; Luo, S.; He, Y.; Han, H.; Raza, F.; Wu, J.; Ge, L., Injectable pH and redox dual responsive hydrogels based on self-assembled peptides for anti-tumor drug delivery. *Biomater Sci* **2020,** *8* (19), 5415-5426.
2. Ghosh, G.; Barman, R.; Sarkar, J.; Ghosh, S., pH-Responsive Biocompatible Supramolecular Peptide Hydrogel. *The Journal of Physical Chemistry B* **2019,** *123* (27), 5909-5915.
3. Lin, B. F.; Megley, K. A.; Viswanathan, N.; Krogstad, D. V.; Drews, L. B.; Kade, M. J.; Qian, Y.; Tirrell, M. V., pH-responsive branched peptide amphiphile hydrogel designed for applications in regenerative medicine with potential as injectable tissue scaffolds. *Journal of Materials Chemistry* **2012,** *22* (37), 19447-19454.
4. Hatip Koc, M.; Cinar Ciftci, G.; Baday, S.; Castelletto, V.; Hamley, I. W.; Guler, M. O., Hierarchical Self-Assembly of Histidine-Functionalized Peptide Amphiphiles into Supramolecular Chiral Nanostructures. *Langmuir* **2017,** *33* (32), 7947-7956.
5. Raza, F.; Zhu, Y.; Chen, L.; You, X.; Zhang, J.; Khan, A.; Khan, M. W.; Hasnat, M.; Zafar, H.; Wu, J.; Ge, L., Paclitaxel-loaded pH responsive hydrogel based on self-assembled peptides for tumor targeting. *Biomaterials Science* **2019,** *7* (5), 2023-2036.
6. Kaur, H.; Sharma, P.; Patel, N.; Pal, V. K.; Roy, S., Accessing Highly Tunable Nanostructured Hydrogels in a Short Ionic Complementary Peptide Sequence via pH Trigger. *Langmuir* **2020,** *36* (41), 12107-12120.
7. Liu, Y.; Ran, Y.; Ge, Y.; Raza, F.; Li, S.; Zafar, H.; Wu, Y.; Paiva-Santos, A. C.; Yu, C.; Sun, M.; Zhu, Y.; Li, F., pH-Sensitive Peptide Hydrogels as a Combination Drug Delivery System for Cancer Treatment. *Pharmaceutics* **2022,** *14* (3).
8. Yamamoto, S.; Nishimura, K.; Morita, K.; Kanemitsu, S.; Nishida, Y.; Morimoto, T.; Aoi, T.; Tamura, A.; Maruyama, T., Microenvironment pH-Induced Selective Cell Death for Potential Cancer Therapy Using Nanofibrous Self-Assembly of a Peptide Amphiphile. *Biomacromolecules* **2021,** *22* (6), 2524-2531.
9. Schneider, J. P.; Pochan, D. J.; Ozbas, B.; Rajagopal, K.; Pakstis, L.; Kretsinger, J., Responsive Hydrogels from the Intramolecular Folding and Self-Assembly of a Designed Peptide. *Journal of the American Chemical Society* **2002,** *124* (50), 15030-15037.
10. Dexter, A. F.; Fletcher, N. L.; Creasey, R. G.; Filardo, F.; Boehm, M. W.; Jack, K. S., Fabrication and characterization of hydrogels formed from designer coiled-coil fibril-forming peptides. *RSC Advances* **2017,** *7* (44), 27260-27271.
11. Liu, Y.; Yang, Y.; Wang, C.; Zhao, X., Stimuli-responsive self-assembling peptides made from antibacterial peptides. *Nanoscale* **2013,** *5* (14), 6413-21.
12. Bortolini, C.; Liu, L.; Hoffmann, S. V.; Jones, N. C.; Knowles, T. P. J.; Dong, M., Exciton Coupling of Phenylalanine Reveals Conformational Changes of Cationic Peptides. *ChemistrySelect* **2017,** *2* (8), 2476-2479.
13. Decandio, C. C.; Silva, E. R.; Hamley, I. W.; Castelletto, V.; Liberato, M. S.; Oliveira, V. X.; Oliveira, C. L. P.; Alves, W. A., Self-Assembly of a Designed Alternating Arginine/Phenylalanine Oligopeptide. *Langmuir* **2015,** *31* (15), 4513-4523.
14. Perween, S.; Chandanshive, B.; Kotamarthi, H. C.; Khushalani, D., Single amino acid based self-assembled structure. *Soft Matter* **2013,** *9* (42), 10141-10145.

15. Krysmann, M. J.; Castelletto, V.; Kelarakis, A.; Hamley, I. W.; Hule, R. A.; Pochan, D. J., Self-Assembly and Hydrogelation of an Amyloid Peptide Fragment. *Biochemistry* **2008,** *47* (16), 4597-4605.
